# Supplementary material for: Efficacy and effectiveness of COVID-19 vaccines in Africa: A systematic review
Source: PLoS One. 2024 Jun 28;19(6):e0306309. doi: 10.1371/journal.pone.0306309 (PMC11213354; doi:10.1371/journal.pone.0306309)
Supplement: S1 Table — (DOCX) [file pone.0306309.s002.docx]

**S1 Table: Database Search Strategy**

| **Source of literature** | **COVID-19 Vaccination terms** | **Effectiveness terms** | **Africa terms** |
| --- | --- | --- | --- |
| PUBMED* | COVID-19 [Title/Abstract] OR Coronavirus disease [Title/Abstract] OR SARS-COV-2 [Title/Abstract] OR Vaccine [Title/Abstract] OR Vaccination [Title/Abstract] | Effectiveness [mh] OR Effectiveness [Title/Abstract] OR Efficiency [Title/Abstract] OR Efficiency [Title/Abstract] OR Effect [Title/Abstract] | Africa[mh] OR Africa*[tiab] OR Algeria*[tiab] OR Angola*[tiab] OR Benin*[tiab] OR Botswana*[tiab] OR Burkina Faso [tiab] OR Burundi*[tiab] OR Cape Verde*[tiab] OR Cabo Verde [tiab] OR Cameron*[tiab] OR Cameroon*[tiab] OR Chad*[tiab] OR Comoros*[tiab] OR Congo*[tiab] OR Cote d'Ivoire[tiab] OR Ivory coast [tiab] OR Djibouti*[tiab] OR Egypt*[tiab] OR Eritrea*[tiab] OR Ethiopia*[tiab] OR Gabon*[tiab] OR Gambia*[tiab] OR Ghana*[tiab] OR Guinea*[tiab] OR Kenya*[tiab] OR Lesotho*[tiab] OR Liberia*[tiab] OR Libya*[tiab] OR Madagascar*[tiab] OR Malawi*[tiab] OR Mali*[tiab] OR Maurit*[tiab] OR Morocc*[tiab] OR Mozambiqu*[tiab] OR Namibia*[tiab] OR Niger*[tiab] OR Rwanda*[tiab] OR Senegal*[tiab] OR Seychelles[tiab] OR Sierra Leone*[tiab] OR Somalia*[tiab] OR Sudan*[tiab] OR Swaziland*[tiab] OR Tanzania*[tiab] OR Togo*[tiab] OR Tunisia*[tiab] OR Uganda*[tiab] OR Zambia*[tiab] OR Zimbabwe*[tiab] |
| Scopus | TITLE-ABS-KEY [COVID-19 OR Coronavirus disease OR SARS-COV-2 OR Vaccine OR Vaccination] | TITLE-ABS-KEY [Effectiveness OR Efficiency OR Effect] | TITLE-ABS-KEY [Africa OR Algeria OR Angola OR Benin OR Botswana OR Burkina Faso OR Burundi OR Cape Verde OR Cabo Verde ORCameron OR Cameroon OR Chad OR Comoros OR Congo OR Cote d’Ivoire OR Ivory Coast OR Djibouti OR EgyptOR Eritrea OR Ethiopia OR Gabon OR Gambia OR Ghana OR Guinea OR Kenya OR Lesotho OR Liberia OR Libya OR Madagascar OR Malawi OR Mali OR Maurit OR Morocco OR Mozambique OR Namibia OR Niger OR Rwanda OR Senegal OR Seychelles OR Sierra Leone OR Somalia OR Sudan OR Swaziland OR Tanzania OR Togo OR Tunisia OR Uganda OR Zambia OR Zimbabwe] |
| Google Scholar | COVID-19 vaccines OR COVID-19 vaccination OR Coronavirus disease vaccines OR Coronavirus disease vaccination OR SARS-COV-2 Vaccine OR SARS-COV-2 Vaccination | Effectiveness OR Efficiency OR Effect | Africa OR Algeria OR Angola OR Benin OR Botswana OR Burkina Faso OR Burundi OR Cape Verde OR Cabo Verde ORCameron OR Cameroon OR Chad OR Comoros OR Congo OR Cote d'Ivoire OR, Ivory Coast OR Djibouti OR EgyptOR Eritrea OR Ethiopia OR Gabon OR Gambia OR Ghana OR Guinea OR Kenya OR Lesotho OR Liberia OR Libya OR Madagascar OR Malawi OR Mali OR Maurit OR Morocco OR Mozambique OR Namibia OR Niger OR Rwanda OR Senegal OR Seychelles OR Sierra Leone OR Somalia OR Sudan OR Swaziland OR Tanzania OR Togo OR Tunisia OR Uganda OR Zambia OR Zimbabwe) |

***Search terms used in PubMed**

((COVID-19 [Title/Abstract] OR Coronavirus disease [Title/Abstract] OR SARS-COV-2 [Title/Abstract] AND Vaccine [Title/Abstract] AND Vaccination [Title/Abstract]) AND (Effectiveness [mh] OR Effectiveness [Title/Abstract] OR Efficiency [Title/Abstract] OR Efficiency [Title/Abstract] OR Efficacy [Title/Abstract] OR Effect [Title/Abstract])) AND (Africa[mh] OR Africa*[tiab] OR Algeria*[tiab] OR Angola*[tiab] OR Benin*[tiab] OR Botswana*[tiab] OR Burkina Faso [tiab] OR Burundi*[tiab] OR Cape Verde*[tiab] OR Cabo Verde [tiab] OR Cameron*[tiab] OR Cameroon*[tiab] OR Chad*[tiab] OR Comoros*[tiab] OR Congo*[tiab] OR Cote d'Ivoire[tiab] OR Ivory coast [tiab] OR Djibouti*[tiab] OR Egypt*[tiab] OR Eritrea*[tiab] OR Ethiopia*[tiab] OR Gabon*[tiab] OR Gambia*[tiab] OR Ghana*[tiab] OR Guinea*[tiab] OR Kenya*[tiab] OR Lesotho*[tiab] OR Liberia*[tiab] OR Libya*[tiab] OR Madagascar*[tiab] OR Malawi*[tiab] OR Mali*[tiab] OR Maurit*[tiab] OR Morocc*[tiab] OR Mozambiqu*[tiab] OR Namibia*[tiab] OR Niger*[tiab] OR Rwanda*[tiab] OR Senegal*[tiab] OR Seychelles[tiab] OR Sierra Leone*[tiab] OR Somalia*[tiab] OR Sudan*[tiab] OR Swaziland*[tiab] OR Tanzania*[tiab] OR Togo*[tiab] OR Tunisia*[tiab] OR Uganda*[tiab] OR Zambia*[tiab] OR Zimbabwe*[tiab])
