# Supplementary material for: Efficacy and effectiveness of COVID-19 vaccines in Africa: A systematic review
Source: PLoS One. 2024 Jun 28;19(6):e0306309. doi: 10.1371/journal.pone.0306309 (PMC11213354; doi:10.1371/journal.pone.0306309)
Supplement: S2 Table — (DOCX) [file pone.0306309.s003.docx]

**S2 Table: Summary of quality assessments using JBI appraisal checklist**

| **S.No** | **Authors, year** | **Study design** | **Items on** **Joanna Briggs Institute** | | | | | | | | | | | | | **Raw score and %** | **Risk** |
| --- | --- | --- | --- | --- | --- | --- | --- | --- | --- | --- | --- | --- | --- | --- | --- | --- | --- |
|  |  |  | **Q1** | **Q2** | **Q3** | **Q4** | **Q5** | **Q6** | **Q7** | **Q8** | **Q9** | **Q10** | **Q11** | **Q12** | **Q13** |  |  |
| 1 | Shinde V, et al, 2022 | RCT | 1 | 1 | 1 | 1 | 1 | 1 | U | 1 | 1 | 1 | 1 | 1 | 1 | 12/13=  92.31% | Low |
| 2 | Madhi S, et.al., 2022 | RCT | 1 | 1 | 1 | 1 | 1 | 1 | U | 1 | 1 | 1 | 1 | 1 | 1 | 12/13=  92.31% | Low |
| 3 | Hardt K, et al., 2021 | RCT | 1 | 1 | 1 | 1 | 1 | 1 | U | 1 | 1 | 1 | 1 | 1 | 1 | 12/13=  92.31% | Low |
| 4 | Sadoff J, et al., 2021 | RCT | 1 | 1 | 1 | 1 | 1 | 0 | U | 1 | 1 | 1 | 1 | 1 | 1 | 11/13=  84.62% | Low |
| 5 | Moreira E, et al., 2022 | RCT | 1 | 1 | 1 | 0 | 1 | 1 | U | 1 | 1 | 1 | 1 | 1 | 1 | 11/13=  84.62% | Low |
| 6 | Bekker L, et al., 2022 | Single arm trial | 0 | 0 | 1 | 1 | 1 | 1 | U | 1 | 1 | 1 | 1 | 1 | 1 | 10/13 = 76.9% | Low |
| 7 | Thomas S, et al., 2021 | Single arm trial | 0 | 0 | 1 | 0 | 1 | 1 | U | 1 | 1 | 1 | 1 | 1 | 1 | 9/13 = 76.9% | Low |

**Summary of quality assessments using JBI appraisal checklist**

| **S.No** | **Authors, year** | **Study design** | **Items on Joanna Briggs Institute** | | | | | | | | | | | **Raw score and %** | **Risk** |
| --- | --- | --- | --- | --- | --- | --- | --- | --- | --- | --- | --- | --- | --- | --- | --- |
|  |  |  | **Q1** | **Q2** | **Q3** | **Q4** | **Q5** | **Q6** | **Q7** | **Q8** | **Q9** | **Q10** | **Q11** |  |  |
| 1 | Ashmawy R, et al., 2023 | Prospective cohort study | 1 | 1 | 1 | 1 | 1 | 1 | 1 | 1 | 1 | 0 | 1 | 10/11=90.9% | Low |
| 2 | Chanda D, et al., 2022 | Retrospective cohort study | 1 | 1 | 1 | 1 | 0 | 1 | 1 | 0 | 1 | 0 | 1 | 8/11 = 72.7% | Low |
| 3 | Zhang Y, et al., 2022 | Retrospective cohort study | 1 | 1 | 1 | 1 | 0 | 1 | 1 | 1 | 1 | 0 | 1 | 9/11 = 81.8% | Low |

**Summary of quality assessments using JBI appraisal checklist.**

| **S.No** | **Authors, year** | **Study design** | **Items on Joanna Briggs Institute** | | | | | | | | | | **Raw score and %** | **Risk** |
| --- | --- | --- | --- | --- | --- | --- | --- | --- | --- | --- | --- | --- | --- | --- |
|  |  |  | **Q1** | **Q2** | **Q3** | **Q4** | **Q5** | **Q6** | **Q7** | **Q8** | **Q9** | **Q10** |  |  |
| 1 | Gray G, et al., 2022 | Test negative case-control study | 1 | 1 | 1 | 1 | 1 | 1 | 1 | 1 | 0 | 1 | 9/10 = 90% | Low |
| 2 | Belayachi J, et al., 2022 | Test negative case-control study | 1 | 1 | 1 | 1 | 0 | 1 | 1 | 1 | 0 | 1 | 8/10 = 80% | Low |
| 3 | Simwanza J, et al., 2021 | Case-control study | 1 | 1 | 1 | 1 | 1 | 0 | 0 | 1 | 0 | 1 | 7/10 = 70% | Low |
